# Transcriptional Regulation and Signaling of Developmental Programmed Cell Death in Plants

**DOI:** 10.3389/fpls.2021.702928

**Published:** 2021-07-29

**Authors:** Cheng Jiang, Jiawei Wang, Hua-Ni Leng, Xiaqin Wang, Yijing Liu, Haiwen Lu, Meng-Zhu Lu, Jin Zhang

**Affiliations:** ^1^State Key Laboratory of Subtropical Silviculture, College of Forestry and Biotechnology, Zhejiang A&F University, Hangzhou, China; ^2^Department of Horticultural Science, North Carolina State University, Raleigh, NC, United States

**Keywords:** programmed cell death, signaling, transcriptional regulation, plant development, cell differentiation

## Abstract

Developmental programmed cell death (dPCD) has multiple functions in plant growth and development, and is of great value for industrial production. Among them, wood formed by xylem dPCD is one of the most widely used natural materials. Therefore, it is crucial to explore the molecular mechanism of plant dPCD. The dPCD process is tightly regulated by genetic networks and is involved in the transduction of signaling molecules. Several key regulators have been identified in diverse organisms and individual PCD events. However, complex molecular networks controlling plant dPCD remain highly elusive, and the original triggers of this process are still unknown. This review summarizes the recent progress on the transcriptional regulation and signaling of dPCD during vegetative and reproductive development. It is hoped that this review will provide an overall view of the molecular regulation of dPCD in different developmental processes in plants and identify specific mechanisms for regulating these dPCD events. In addition, the application of plants in industrial production can be improved by manipulating dPCD in specific processes, such as xylogenesis.

## Introduction

Programmed cell death (PCD) is considered a behavior of self-salvage that is genetically controlled to eliminate no longer needed and damaged cells selectively or to differentiate specific cell types for efficient utilization of nutrition, reproduction, and other aspects (Daneva et al., 2016). In plants, PCD can be classified into developmentally induced PCD (dPCD, triggered as the ultimate step of cell-type specific differentiation programs) and environmentally induced PCD (ePCD, triggered by diverse abiotic stresses) according to the way it is triggered (Petrov et al., 2015; Huysmans et al., 2017). In an early work, PCD and autolysis were considered to be interchangeable or distinct in various studies. However, in some cases of developmental cell death, PCD and autolysis can easily be distinguished as two different biological processes with unique biochemical mechanisms and functional purposes (Escamez and Tuominen, 2014). dPCD occurs during vegetative and reproductive development and is the final differentiation process in specific cell types, such as xylem, root cap cells, trichomes, and anther tapetum (Olvera-Carrillo et al., 2015).

Over the last two decades, numerous studies unscrambling dPCD have focused on (1) the extracellular and intracellular signals that initiate dPCD or are altered during dPCD (Gechev and Hille, 2005), (2) the transcriptional regulation of specific gene expression to control dPCD (Cubría-Radío and Nowack, 2019), and (3) hydrolytic enzymes such as proteases and nucleases, which are key executors of dPCD (Buono et al., 2019). The aim of this review is to shed light on the most common instances of dPCD in plant development, focusing on the molecular regulation of the dPCD process, to discuss future research directions contributing to a clear understanding of the mechanisms of dPCD and to take full advantage of dPCD in plants to improve the efficiency of production.

## Transcriptional Regulation of dPCD

### Transcriptional Regulation of Reproductive dPCD

Reproductive growth is a critical process for plant population survival and genetic diversity. Recently, Wang et al. (2021) summarized the regulatory framework of flower-specific dPCD such as tapetal degeneration, pollen tube rupture, transmitting tract formation, and self-incompatibility. Here, we focus on the transcriptional regulation of dPCD in male and female germ cell formation, and fertilization and seed development.

#### Male Germ Cell Formation

**FIGURE 1:**
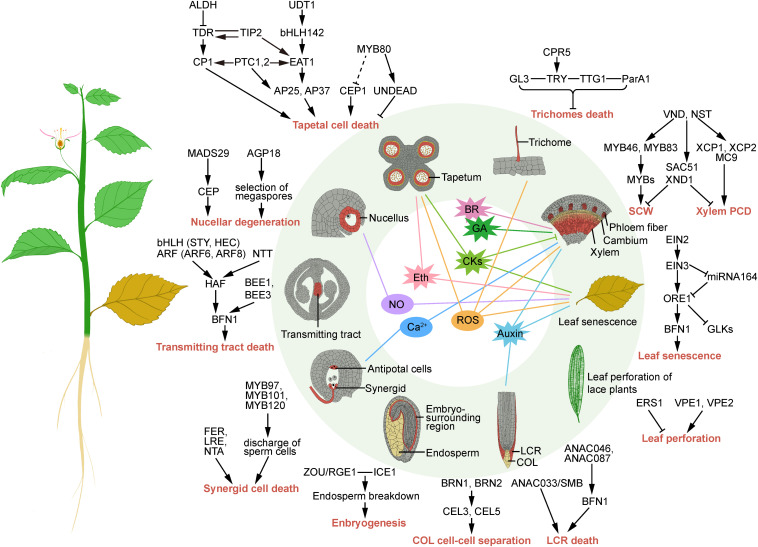
Schematic representation of the transcriptional regulation and signaling of developmental programmed cell death in plants. Arrows and a “T” at the ends of lines represent transcriptional activation and repression, respectively. Lines without arrows or a “T” represent protein-protein interactions; dotted line represents unclear regulation.

Dehiscence of the anther and release of pollen rely on the PCD of the tapetum, and the precise timing of its degeneration is crucial for pollen maturation (Gómez et al., 2015). The transcriptional regulation mechanism that controls dPCD during tapetum differentiation has been reported. A group of basic helix–loop–helix (bHLH) transcription factors (TFs) can positively regulate tapetal PCD. bHLH142 acts downstream of the bHLH TF UNDEVELOPED TAPETUM1 (OsUDT1) and regulates the bHLH TF *ETERNAL TAPETUM1* (*OsEAT1*) to induce tapetal PCD. Furthermore, two genes (*AP25* and *AP37*) that encode aspartic proteases for tapetal PCD are under the regulation of OsEAT1 (Ko et al., 2014). In rice, TAPETUM DEGENERATION RETARDATION (OsTDR) activates a cysteine protease-encoding gene (*OsCP1*) to induce tapetal PCD, and its activation is repressed by an aldehyde dehydrogenase (OsALDH) (Xie et al., 2020). TDR INTERACTING PROTEIN2 (OsTIP2) can interact with and directly regulate the expression of *OsTDR* and *OsEAT1* (Fu et al., 2014). PERSISTENT TAPETAL CELL encoding genes (*OsPTC1* and *OsPTC2*) were proved to regulate the expression of *OsEAT1*, *OsAP1*, and *OsAP25* (Uzair et al., 2020). *Arabidopsis AtMYB80* might suppress tapetal PCD by influencing the expression of the papain-like cysteine protease gene *AtCEP1*, but the precise regulation mechanism is still unclear (Zhang et al., 2014). In addition, AtMYB80 might activate the PCD-inhibiting aspartic protease *UNDEAD*, and a mutation in *AtMYB80* caused precocious tapetum degeneration in *Arabidopsis* (Phan et al., 2011). These results show that a regulatory network is crucial for the precise timing of tapetal PCD and male fertility (Figure 1).

#### Female Germ Cell Formation

Female gametophytes develop inside the ovules, which originate from a meiotic product. One functional megaspore survives, and three megaspores degenerate. During these processes, the expression of *Arabinogalactan protein 18* (*AtAGP18*) is needed in the abaxial pole of the ovule to ensure the survival of functional megaspores (Demesa-Arévalo and Vielle-Calzada, 2013). The nucellus is an ephemeral tissue whose degeneration is controlled by female gametophytes (Chen et al., 2014). The TF OsMADS29 can regulate the expression of the cysteine protease gene (*CEP*), thereby promoting nucellar degeneration in rice (Yin and Xue, 2012). Another PCD-related *CEP* gene, a *vacuolar processing enzyme* (*HvVPE*), was shown to be upregulated during nucellar cell death in barley (Radchuk et al., 2011).

#### Fertilization and Seed Development

During fertilization, several cells are tightly controlled for elimination. Pollen tubes growth toward the ovule is guided by the transmitting tract, the death of which is considered to be important. Cell death of the transmitting tract to make way for growing pollen tubes is regulated by two bHLH TFs (*AtSTY* and *AtHEC*) and two auxin response factor (ARF) genes (*AtARF6* and *AtARF8*) (Kaminaka et al., 2006). Once pollen tubes arrive at ovules, synergids undergo cell death, and three synergid-expressed genes, *FERONIA* (*AtFER*), *LORELEI* (*AtLRE*), and *NORTIA* (*AtNTA*), are required (Kessler and Grossniklaus, 2011). AtMYB97, AtMYB101, and AtMYB120 positively regulate receptive synergid degeneration after the proper discharge of sperm cells (Liang et al., 2013). ZHOUPI (AtZOU/AtRGE1) and INDUCER OF CBP EXPRESSION 1 (AtICE1) promote the breakdown of the embryo-surrounding region, and loss-of-function mutants of these genes lead to misshaped embryos (Denay et al., 2014).

### Transcriptional Regulation of Vegetative dPCD

#### Xylogenesis

Secondary xylem is the most abundant biomass produced by plants and concurrently functions in structural and mechanical support and transport tissues distributing water and solutes. Xylogenesis consists of xylem generation, differentiation, and PCD processes (Escamez and Tuominen, 2014). In this process, a typical dPCD event is the formation of the tracheal element (TE), which loses its contents and forms a functional region composed of hollow, dead tubes. VASCULAR NAC DOMAIN (VNDs) and NAC SECONDARY WALL THICKENING PROMOTING FACTOR (NSTs) are known to function as key regulators of TE secondary cell wall (SCW) formation and cell death (Zhang et al., 2018a). These NAC TFs are regarded as first layer regulators in the progression of the SCW regulatory network, subsequently regulating the second layer master switches *MYB46* and *MYB83* (Zhong et al., 2007). VNDs also regulate the expression of TE differentiation inhibitors, such as *SUPPRESSOR OF ACAULIS 51* (*SAC51*) and *XYLEM NAC DOMAIN 1* (*XND1*) (Zhong et al., 2010). Moreover, hydrolytic enzymes downstream of VNDs, such as xylem cysteine proteinase (AtXCP1 and AtXCP2) and metacaspase9 (AtMC9), are the executors of autolysis during dPCD.

#### Root Cap

The root cap ensheathes the root and has important functions for root growth, gravity sensing, root system architecture, and protection of the stem cells in the root tip (Kumpf and Nowack, 2015). The root cap has to maintain its size and position at the root tip by sloughing old cells and producing new cells; the disposal of old cells is a PCD process. In *Arabidopsis*, the root cap can be distinguished into two tissue types, the central columella root cap (COL) and the peripheral lateral root cap (LRC). Two NACs, BEARSKIN1 (AtBRN1) and AtBRN2, regulate root cap-expressed *CELLULASE3* (*AtCEL3*) and *AtCEL5*, and control cell-cell separation in mature COL layers (Bennett et al., 2010). The TF ANAC033/SOMBRERO was shown to transcriptionally control LRC differentiation and preparation for cell death (Fendrych et al., 2014). In addition, two other NACs (AtANAC087 and AtANAC046) are sufficient to activate the expression of cell death-associated genes and induce dPCD *via* the nuclease BIFUNCTIONAL NUCLEASE1 (AtBFN1) (Huysmans et al., 2018).

#### Leaf Morphogenesis

The production of various leaf shapes during leaf morphogenesis is also regulated by dPCD. Leaf perforation exists in lace plants, and few aroids are caused by PCD (Gunawardena, 2008). The transcription level of the ethylene receptor gene *AmERS1a* was significantly reduced in leaves undergoing PCD, indicating that ethylene plays an important role in dPCD during lace plant perforation (Rantong et al., 2015). Two *VPE* genes (*AmVPE1* and *AmVPE2*) were identified with high expression levels in the preperforation developmental stage and late window stage of lace plants, respectively (Rantong and Gunawardena, 2018). However, the detailed transcriptional regulation mechanism is still unclear.

#### Leaf Senescence

The last stage of leaf senescence is a PCD process influenced by numerous internal and external environmental signals, which are controlled by a very complex gene regulatory network (Woo et al., 2019). Ethylene controls the leaf senescence PCD by the trifurcate pathway. ETHYLENE-INSENSITIVE2 (AtEIN2) perceiving ethylene activates the expression of *AtEIN3*, which represses *miR164* (Li et al., 2013). *MiR164* subsequently suppresses the expression of *ORESARA1/ANAC092 (AtORE1)*, which directly contributes to controlling AtBFN1 (Matallana-Ramirez et al., 2013), and inhibits the activity of the chloroplast-supporting TFs GOLDEN-LIKE1 (AtGLK1) and AtGLK2 (Rauf et al., 2013).

#### Trichome Differentiation

Trichomes are epidermal outgrowths that play various protective roles and provide valuable resources for plant development. Trichome cells were proved to die finally with chromatin condensation, nuclear fragmentation, and endoplasmic reticulum dilation (Papini et al., 2010). Trichome death is also regulated by *CONSTITUTIVE PATHOGEN RESPONSE5* (*AtCPR5*) through linking endoreduplication and cell division (Kirik et al., 2001), and the effects of the *cpr5* mutant were epistatic to those of trichome developmental regulators TRIPTYCHON (AtTRY) and GLABRA3 (AtGL3) (Brininstool et al., 2008). In *Nicotiana*, silencing *TRANSPARENT TESTA GLABRA1* (*NtTTG1*) in trichomes resulted in the elimination of hypersensitive cell death induced by ParA1 (Wang et al., 2009).

## Signals Controlling Developmental PCD

### Plant Hormones

Auxin was reported to be involved in xylem formation, root cap morphogenesis, and leaf senescence. Dying cells were shown to facilitate the production of indole-3-acetic acid (IAA), as their proteins were hydrolyzed and some of the tryptophan was released to adjacent cells (Sheldrake, 2021). The auxin dynamics among dying cells and adjacent cells play a crucial role in plant growth and development through PCD. The levels of auxin immediately increase around differentiating xylem cells in leaf veins, petals, and roots when they are dying (Brunoud et al., 2012). When polar auxin transport is inhibited, xylem differentiation is further induced locally, along with high levels of auxin (Ravichandran et al., 2020). In this process, differentiating xylem cells were considered “stem-cell organizers” leading to the formation of a new organizer adjacent to cambial stem cells and differentiation into mature xylem themselves. In the lateral root cap, PCD and auxin form a cyclic action that regulates the periodicity of lateral organ induction, coordinating primary root growth with root branching (Xuan et al., 2016). Furthermore, in senescent leaves, tryptophan and auxin levels increase dramatically, which accelerates leaf senescence (Araújo et al., 2011). These studies indicate that auxin acts as an important signal involved in cell differentiation, especially in developmentally controlled cell death. Furthermore, it mainly appears to initiate these processes (Vanneste and Friml, 2009).

Cytokinin plays a central role in cell division and differentiation through an antagonistic interaction with auxin. Cytokinin can also affect the dPCD of xylem, leaves during senescence, and tapetum. In *Arabidopsis*, the cell fate to protoxylem exhibits high auxin and low cytokinin signaling, while the cell fate to procambium exhibits high cytokinin but low auxin signaling in roots (Bishopp et al., 2011). In a loss-of-function mutant of the cytokinin receptor *AtAHK3*, leaf senescence was delayed with reduced sensitivity to cytokinin (Kim et al., 2006). Through expression analysis of phytohormone biosynthesis and signaling genes, cytokinin is needed during earlier stages of rice tapetum and not needed at the uninuclear microspore stage (Hirano et al., 2008). Therefore, regulating cytokinin at low levels ensures well-organized PCD in plants. It has also been demonstrated that high levels of cytokinin call induce PCD in root cortex cells of *Vicia faba* ssp. minor seedlings (Kunikowska et al., 2013) and in cultured *Arabidopsis* cells (Vescovi et al., 2012).

Ethylene is widely known as the plant hormone responsible for a number of developmental processes. Tapetal cell death and leaf senescence require ethylene. The reduction of ethylene production and signal transduction leads to delayed leaf senescence (Graaff et al., 2006). Some plants form aerenchyma in response to a hypoxic environment; however, blocking the ethylene receptor that makes the cells insensitive to ethylene can avoid PCD to allow aerenchyma to form (Liu et al., 2019). Ethylene signaling is needed for the induction of PCD in epidermal cells of deepwater rice (Steffens and Sauter, 2005). The expression and activation of signaling genes involved in ethylene specifically occurred in tapetal cells concurrently, which regulated tapetal cell death (Hirano et al., 2008). In addition, ethylene is also an active signal in floral organ formation and development (Wang et al., 2010).

Moreover, several other phytohormones are also involved in dPCD-related processes, e.g., gibberellin is essential in vascular differentiation (Eriksson et al., 2000), brassinosteroids initiate TE differentiation (Milhinhos and Miguel, 2013), salicylic acid is associated with hypersensitive reaction and has dual functions in cell death control (Radojičić et al., 2018), and jasmonic acid is necessary for leaf senescence (He et al., 2002). In summary, phytohormones play many roles in plant dPCD. However, the molecular and biochemical mechanisms of plant hormones as signals for the dPCD of different tissues and organs remain to be elucidated (Figure 1).

### Reactive Oxygen Species

Continuously generated reactive oxygen species (ROS), such as singlet oxygen (^1^O_2_), superoxide (O_2_^.–^), hydrogen peroxide (H_2_O_2_), and hydroxyl radical (HO), act as signaling molecules that coordinate various plant processes, namely, tapetal cell death, trichome death, xylogenesis, and leaf senescence (Gechev et al., 2006). ROS are regarded not only as signals but also as byproducts of aerobic pathways generated in different cellular compartments and cause PCD at higher concentrations (Gechev and Hille, 2005). MAPKs involved in relaying the H_2_O_2_ signal mediate PCD triggered by chloroplast-generated H_2_O_2_ (Liu et al., 2007). The chloroplast protein EXECUTER1, acting together with EXECUTER2, could transfer oxidative signals from the plastid to the nucleus, leading to cell death (Lee et al., 2007). The H_2_O_2_ signal transmitted to ROS-specific TFs could lead to H_2_O_2_-dependent cell death (Gadjev et al., 2006). In the rice *mads3* mutant, anthers exhibit oxidative stress-related phenotypes, since tapetal PCD occurs prematurely (Hu et al., 2011). ROS production by NADPH oxidases also acts on tapetal PCD progression (Xie et al., 2014). Elimination of H_2_O_2_ through non-enzymatic and enzymatic pathways maintains the level of ROS, while catalase, peroxidase, ascorbate peroxidase, and glutathione reductase are considered to be the main H_2_O_2_ scavengers, which can effectively reduce the amount of H_2_O_2_ (Kapoor et al., 2015). In summary, the role of ROS in the dPCD process strictly depends on its concentration, and its interaction with other signaling molecules also determines cell fate (Niu and Liao, 2016).

### Nitric Oxide

Nitric oxide (NO) is a small gaseous and highly reactive molecule that can take part in a wide range of physiological processes, such as nucellar degeneration and leaf senescence. Due to NO being highly reactive, a series of NO derivatives could be formed, resulting in a redox-mediated modification in plants. The effects of NO as an activator or repressor seem to be due to the concentration and timing patterns of NO (Mur et al., 2012). High levels of NO are associated with the progression of natural senescence, cell death, and DNA fragmentation (Carimi et al., 2005). Studies have indicated that NO and H_2_O_2_ can be induced to synthesize each other and that the signaling crosstalk between H_2_O_2_ and NO synergistically regulates leaf cell death and delays senescence (Iakimova and Woltering, 2015). During nucellar cell degeneration, considerable production of NO and H_2_O_2_ caused an induction of caspase-like proteases, leading to nucellar dPCD (Lombardi et al., 2010). In addition, rapid and transient increases in ROS and NO were also detected by self-incompatibility in the pollen tube growth of *Papaver* (Wilkins et al., 2011).

### Calcium

Ca^2+^ is a core signaling molecule in plants that is involved in many physiological processes, such as PCD of synergids and xylogenesis (Uslu and Grossmann, 2016). The change in intracellular Ca^2+^ concentration plays an important role in signaling transmission during a series of cellular processes (Kong et al., 2015). The Ca^2+^ increase in the cytosol was verified to be an early signal that occurred upstream of the vacuolar breakdown in self-incompatibility (Wilkins et al., 2015). Calcium-mediated signaling between two synergids determined their fate (death or survival) in the control of sperm delivery (Ngo et al., 2014). In xylogenic *Zinnia* cultures, Ca^2+^ influx into the cell was required for cell death and implicated as a trigger after SCW deposition (Groover and Jones, 1999). Ca^2+^ influx has been demonstrated to be mediated by H_2_O_2_ signaling in the plasma membrane of root cells, resulting in root elongation (Han et al., 2014). Because of various types of Ca^2+^ receptors and channels, Ca^2+^ signaling can be delivered widely. For example, Ca^2+^-dependent DNases (CaN) participate in the PCD of secretory cavity cells through nuclear DNA degradation in *Citrus* (Bai et al., 2020). In *Eucommia ulmoides*, *EuCaN1*, and *EuCaN2* were identified to be involved in secondary xylem development (Chen et al., 2012).

## Conclusion and Future Perspectives

Current studies have shown that the fate of cells undergoing PCD is controlled by complex signaling and transcriptional regulatory networks. Transcriptional regulation has been revealed to play a key role in specific dPCD events. However, the link between signaling pathways and dPCD-related gene expression regulation is rarely clearly established. Especially in woody plants, the signal transduction and transcriptional regulatory mechanism of dPCD during wood formation are still unclear. Recently, Zhang et al. (2018b) used a combination of expression quantitative trait loci (eQTL) analysis and genome-wide association study (GWAS) to successfully identify crucial genes that control metabolite synthesis and further upstream transcription factors. This approach provides a new strategy for exploring new regulatory mechanisms in biological processes. Therefore, combining cell biology, biochemistry, and molecular biology methods to detect how signaling pathways control dPCD through gene regulatory networks will become an overall trend in future research. Specifically, xylogenesis contributes to the largest bioenergy source, in which the components and contents of SCW are the main influencing factors in the improvement of industrial production. In short, the analysis of the molecular regulation mechanism of plant dPCD can lay the foundation for regulating specific developmental processes of plants through genetic engineering methods and further applying their products to industrial production.

## Author Contributions

JZ conceived the study. CJ and JZ collected and synthesized the data and draft the manuscript. JW, H-NL, XW, YL, HL, and M-ZL revised the manuscript. All authors contributed to the article and approved the submitted version.

## Conflict of Interest

The authors declare that the research was conducted in the absence of any commercial or financial relationships that could be construed as a potential conflict of interest.

## Publisher’s Note

All claims expressed in this article are solely those of the authors and do not necessarily represent those of their affiliated organizations, or those of the publisher, the editors and the reviewers. Any product that may be evaluated in this article, or claim that may be made by its manufacturer, is not guaranteed or endorsed by the publisher.
